# Congenital Complete Atrioventricular Block and Dilated Cardiomyopathy: New Light for an Old Disease

**DOI:** 10.1155/2012/451375

**Published:** 2012-08-02

**Authors:** Mariana Paiva, Vania Ribeiro, Raquel Garcia, Sandra Amorim, Manuel Campelo, Elisabete Martins, Brenda Moura, Jose Cardoso, M. Júlia Maciel

**Affiliations:** Cardiology Department, Centro Hospitalar São João, 4200-319 Porto, Portugal

## Abstract

We present a case of a patient with known complete congenital atrioventricular block (CAVB) since the age of 7 years old that developed dilated cardiomyopathy ten years after VVI-R pacemaker implantation. He presented severe biventricular dysfunction and was symptomatic despite optimal medical therapy. Cardiac resynchronization therapy was used, and he showed clinical and electrocardiographic improvement a month later.

## 1. Case Report

**Figure 1:**
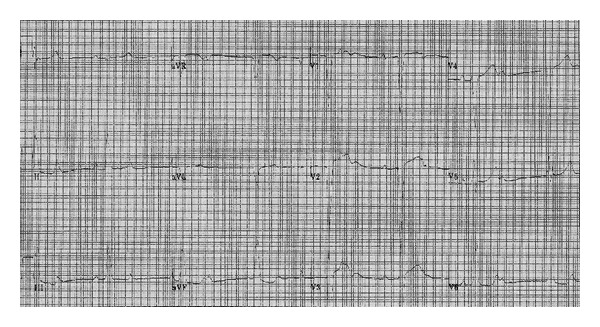
EKG showing complete heart block with ventricular escape rhythm.

A 7-year-old boy was sent, in 1997, to a pediatric cardiology outpatient clinic, due to a slow heart rate and exercise intolerance. His EKG showed sinus rhythm, 44 bpm, complete atrioventricular block (CAVB), and left bundle branch block (Figure 1). His TTE showed no structural abnormalities. Exercise treadmill test revealed an inappropriate chronotropic response to exercise. The 24-hour Holter test confirmed permanent CAVB and the absence of other abnormalities.

**Figure 2:**
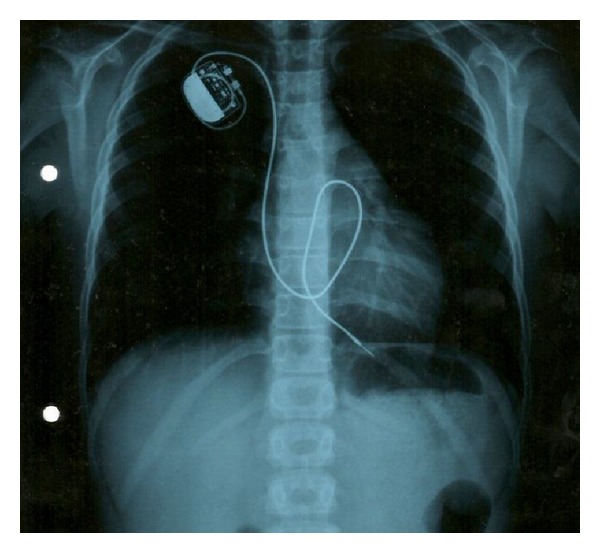
Thorax X-ray after VVI-R implantation.

At age 11, for worsening of exercise tolerance, a VVI-R pacemaker was implanted (Figure 2). He was completely asymptomatic, and by 19 years old the TTE showed a severe LVSD, with slight increase of LV diameter and mild right ventricular systolic dysfunction; he started lisinopril and bisoprolol.

**Figure 3:**
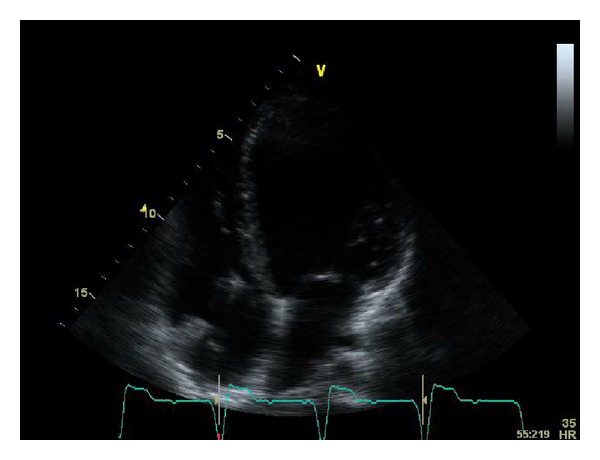
ETT showing dilatation of left ventricle.

By age 21, he became symptomatic in NYHA class IV and hypotensive. BNP and D-dimers levels were high. The EKG was similar to previous ones, and the TTE showed severe right chamber dilatation (LVEDD of 65 mm), severe biventricular dysfunction (LVEF 21%), severe tricuspid regurgitation and estimated PASP of 62 mmHg (Figure 3). A chest CT scan showed signs of chronic pulmonary embolism. Full dose enoxaparin was started, and he was hospitalized.

He improved clinically, although maintaining mild-to-moderate exertion dyspnea. TEE showed no left atrial appendage thrombus, and the genetic prothrombotic analyses revealed a deficiency of C-protein.

**Figure 4:**
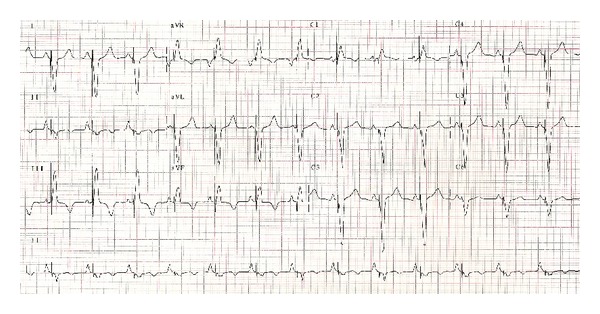
ECG after CRT implantation.

As he was lifelong pacemaker dependent, with severe biventricular dysfunction and being symptomatic despite medical therapy, VVI-R was upgraded to CRT-D. QRS complex became narrower (from 174 to 126 mseg) (Figure 4). He was discharged on candesartan, bisoprolol, spironolactone, warfarin, and furosemide.

One month after, in NYHA class II, TTE showed a slight improvement in left ventricular function, while maintaining right chamber dilation and pulmonary hypertension (PASP 67 mmHg).

## 2. Discussion

This case illustrates the complexity of management of patients with congenital CAVB. The diagnosis can be quite challenging and is more commonly done during childhood, even though a timeline for diagnosis can begin in uterus until late adulthood. The most common signs are bradycardia and inadequate chronotropic response to a stress test. There is a wide variability in symptom presentation from fatigue, exercise intolerance, and the most preoccupating syncope and sudden death. The evolution to dilated cardiomyopathy is well documented and not uncommon [1], although different mechanisms have been proposed and reported. In the case of our patient, the development of pulmonary hypertension due to chronic thromboembolism was a complication factor and certainly an occurrence that would be responsible for a worse outcome. Approximately 40% of patients with protein C deficiency present with evidence of pulmonary embolism, and roughly 60% suffer recurrent thrombosis if anticoagulation is discontinued. Even though recent reports have demonstrated the good overall prognosis associated with CAVB, the presence of maternal autoantibodies (anti-Ro and anti-LLA) [2] at the time of diagnosis has been associated with a worse outcome, and the deleterious effect of right ventricular pacing is still in debate [3, 4]. This sets the discussion on when is the ideal time for pacemaker implantation. Indications for pacemaker implantation are the presence of symptoms, including exercise intolerance, slow ventricular escape rhythms, and previous Stoke-Adams attacks in order to prevent sudden death occurrence. For the first time, in early 2004, the deleterious effects of right ventricular pacing are recognized as a cause for dilated cardiomyopathy, and this adds for the controversy on prophylactic pacemaker in these patients. Despite this acknowledgment, it has been observed that these patients tolerate well lifelong pacing, and the evolution to dilated cardiomyopathy has been related to other etiologies usually related to the disease itself [2].

The implantation of biventricular pacing in some selected cases might be the solution after the tragic development to dilated cardiomyopathy in these patients [3, 5].
